# Genomics and the Acute Respiratory Distress Syndrome: Current and Future Directions

**DOI:** 10.3390/ijms20164004

**Published:** 2019-08-16

**Authors:** Tamara Hernández-Beeftink, Beatriz Guillen-Guio, Jesús Villar, Carlos Flores

**Affiliations:** 1 Research Unit, Hospital Universitario Dr. Negrín, 35010 Las Palmas de Gran Canaria, Spain; 2 Research Unit, Hospital Universitario N.S. de Candelaria, Universidad de La Laguna, 38010 Santa Cruz de Tenerife, Spain; 3 CIBER de Enfermedades Respiratorias, Instituto de Salud Carlos III, 28029 Madrid, Spain; 4 Genomics Division, Instituto Tecnológico y de Energías Renovables (ITER), 38600 Santa Cruz de Tenerife, Spain; 5 Instituto de Tecnologías Biomédicas (ITB), Universidad de La Laguna, 38200 Santa Cruz de Tenerife, Spain

**Keywords:** ARDS, biomarkers, genetic risks, genomics, pathophysiology

## Abstract

The excessive hospital mortality associated with acute respiratory distress syndrome (ARDS) in adults mandates an urgent need for developing new therapies and tools for the early risk assessment of these patients. ARDS is a heterogeneous syndrome with multiple different pathogenetic processes contributing differently in different patients depending on clinical as well as genetic factors. Identifying genetic-based biomarkers holds the promise for establishing effective predictive and prognostic stratification methods and for targeting new therapies to improve ARDS outcomes. Here we provide an updated review of the available evidence supporting the presence of genetic factors that are predictive of ARDS development and of fatal outcomes in adult critically ill patients and that have been identified by applying different genomic and genetic approaches. We also introduce other incipient genomics approximations, such as admixture mapping, metagenomics and genome sequencing, among others, that will allow to boost this knowledge and likely reveal new genetic predictors of ARDS susceptibility and prognosis among critically ill patients.

## 1. Definition and Epidemiology

The acute respiratory distress syndrome (ARDS) is an acute and intense pulmonary inflammatory process caused by pulmonary or systemic insults, most commonly initiated by pneumonia, sepsis or trauma [1]. These lesions trigger a non-specific immune response in the lung, leading to an increase in the permeability of the alveolar-capillary membrane and the formation of alveolar protein-rich oedema. Despite ARDS is rare in the population—its estimated incidence is variable among published studies but can be as few as 7 cases per 100,000 individuals—it continues to be an important cause of death in adult intensive care units (ICUs) (30–40%) [1,2,3]. Furthermore, the development of ARDS has significant long-term consequences among survivors, such as physical and cognitive impairment [4]. Current identification of ARDS lacks a simple diagnostic test and is based on the constellation of diverse clinical and imaging signs that are indicative of acute respiratory failure [1,5]. Depending on the severity of the lung failure, measured by the PaO_2_/FiO_2_ ratio, ARDS is currently classified as mild (200 < PaO_2_/FiO_2_ ≤300), moderate (100 < PaO_2_/FiO_2_ ≤ 200) or severe (PaO_2_/FiO_2_ ≤100) [1].

Since its first description in 1967 [6], there has been a significant progress in elucidating the pathophysiology of this complex syndrome. However, the absence of a universal and homogeneous mechanism of the disease has led to practical problems for providing a definitive treatment [7]. After more than 50 years since its description, the existing treatment strategies for ARDS continue to be based on supportive interventions [8], where mechanical ventilation (MV) remains the main life-saving strategy. Because of that, current trends focus on the early identification and prevention of ARDS development [9,10]. Beyond providing new insights into the pathogenesis, the identification of ARDS biomarkers can help to predict disease susceptibility, to stratify the risk for death and to reveal new therapeutic targets [11]. Here we provide an updated review of the available evidence supporting the existence of genetic factors that are predictive for ARDS development or fatal outcomes among adult ICU patients and the main incipient genomic approaches that hold the promise for important discoveries in this field in the years to come (Table 1).

## 2. Molecular Pathophysiology

The physiopathological manifestations of ARDS are associated with diverse risk factors. Some of the heterogeneity of ARDS risk and outcome may be explained by different underlying biology resulting in a similar clinical presentation. The main hallmark of ARDS is the presence of diffuse alveolar damage characterized by extensive inflammation of the lung tissue, which results in an initial release of molecules and inflammatory mediators by local epithelial and endothelial cells. The injury of type-II alveolar cells and the endothelial activation can lead to an obstruction or destruction of the pulmonary vasculature and to an increase of lung tissue permeability with deposition of proteins and debris in the alveoli and formation of an inflammatory pulmonary oedema [12]. The vascular endothelial growth factor (VEGF), a glycoprotein that intervenes in vascular permeability, has a critical role in the maintenance of normal alveolar structures [13,14,15]. By one hand, VEGF participates in tissue repair after lung injury through epithelial regeneration. As an example, and although it is subject to debate, it has been shown that levels of VEGF in the lungs of ARDS patients are lower than those of the controls, suggesting a role in endothelial cell apoptosis and the capillary density [16]. On the other hand, detrimental effects are also suggested for VEGF as it compromises the integrity of the alveolar-capillary barrier promoting pulmonary oedema. However, VEGF has a complex pleiotropic activity beyond the mere regulation of the alveolar-capillary barrier [17,18]. Therefore, a better understanding of activities of the VEGF family members is still needed to fully understand their role in ARDS [15].

In addition to the endothelial barrier dysfunction, the accumulation of neutrophils in the lungs and the role of certain systemic factors are also central for the pathological endpoint of ARDS [19]. It has been observed that pathogen-associated molecular patterns (PAMPs), such as the lipopolysaccharide (LPS) of Gram-negative bacteria, lead to the activation of several signal pathways responsible for triggering inflammatory mediators that further contribute to lung tissue stress and alveolar-capillary barrier dysfunction [20,21,22]. The inflammation and recruitment of neutrophils through the liberation of mediators such as chemokines, cytokines and growth factors, also induce the activation of cell proliferation and division. Toll-like receptors, which constitute the main PAMP receptors, also recognize molecules that are released with cellular debris by necrotic tissues [23] and induce the expression of inflammatory mediators, causing inflammatory cell infiltration into the alveolar space and, ultimately, contributing to lung inflammation and further impairment of respiratory function. Among many others, cell-free mitochondrial DNA (mtDNA) and mitochondrial peptides are endogenous damage-associated molecular patterns (DAMPs) [24,25], which are also known to play a critical role in modulating the response to pulmonary injury [26,27]. Linked to this, lung injury can be triggered by failures in cell division through diverse proteins related to apoptosis, such as Bax, Bcl-2 and cleaved-caspase-3, which are also known as biomarkers of lung injury [28], as their levels change markedly during the early stages of the disease process [29]. In fact, some studies in experimental animal models have focused on the therapeutic use of microRNA targets to reduce apoptosis and, concomitantly, the levels of inflammatory factors such as interleukin (IL)-1β (IL-1β) and tumour necrosis factor alpha (TNF-α) during experimental ARDS [30].

## 3. Genetic Association Studies

**Figure 1:**
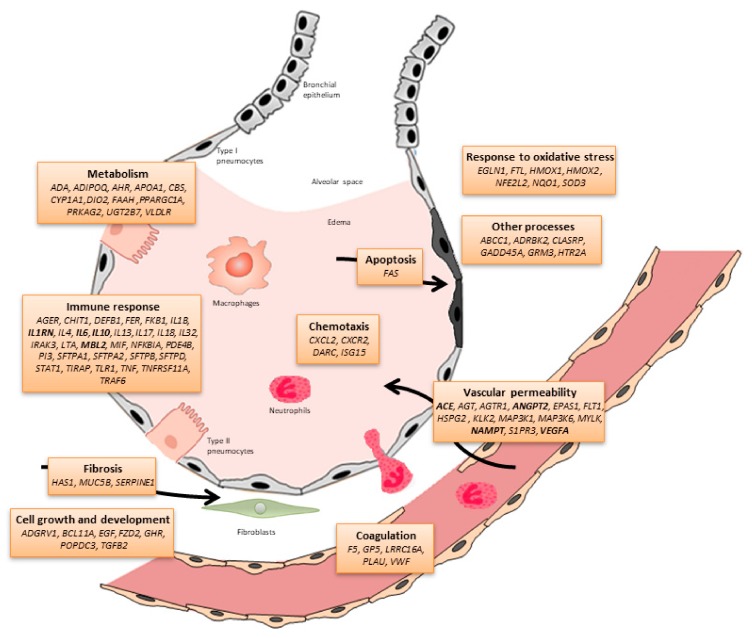
Schematic representation of the alveolar-capillary barrier including the candidate genes (and biological processes) associated with ARDS susceptibility and outcomes to date (modified from Guillen-Guio et al. [33]). Genes associated with ARDS in at least four studies are indicated in bold.

Because of the limited therapeutic options in ARDS, there is an urgent need to identify biomarkers, including genetic factors, that can help to stratify the risks in ICU patients, to predict their prognosis, and, possibly, to serve as more specific therapeutic targets. Many of the genetic studies to date have focused on identifying inherited risk variants in genes encoding biological candidates linked to the immune response, tissue permeability and vascular metabolism, cell growth and development, response to the oxidative stress and to coagulation [31,32,33] (Figure 1).

While there is no doubt that candidate-gene association studies have been questioned due to the lack of reproducibility and the difficulties in their interpretation [34,35], they have helped to point out a few genes that have been associated several times with ARDS susceptibility or outcome in independent studies, suggesting a genuine implication in disease: the interleukin 6 (*IL6*), interleukin 10 (*IL10*), interleukin 1 receptor antagonist (*IL1RN*), vascular endothelial growth factor A (*VEGFA*; also known as VEGF), angiotensin-converting enzyme (*ACE*), soluble mannose-binding lectin 2 (*MBL2*) and visfatin (*NAMPT*) [33].

Until 2015, a total of 81 different candidate genes were associated with ARDS susceptibility or outcomes in 68 independent studies, most of them performed in patients of European ancestry [33]. From 2015 to April 2019, 10 novel candidate genes have been explored in 10 independent studies (Figure 1, Table 2).

In this context, Dötsch and colleagues observed that a variant of the Egl-9 family hypoxia inducible factor 1 (*EGLN1*) gene was independently associated with greater ARDS mortality risk within 30-days in Europeans [36]. EGLN1 and the hypoxia inducible factor degrading prolyl-hydroxylases (PHD) are key regulators of the human response to a low oxygen environment, which provide a link between tissue hypoxia and the inflammatory response [46]. Despite the biological plausibility, further studies should be carried out to assess the functional impact of the genotype in *EGLN1* in the mechanisms leading to ARDS [36]. Consonant to the fact that pulmonary fibrosis develops during the intermediate or late stages of ARDS, another study [37] reported the association of the strongest common risk gene variant for idiopathic pulmonary fibrosis (IPF) known to date [47] with ARDS susceptibility. A polymorphism located in the mucin 5B (*MUC5B*) gene promoter showed clinical relevance and large effect sizes for IPF (odds ratio [OR] > 6.0 per allele) [37]. The authors found that the subjects who were homozygous for the *MUC5B* variant also had a moderate risk for developing ARDS (OR: 1.47; 95%CI: 1.02–2.1). While this finding needs further validation in independent studies, it supports the possibility that there may be shared genetic risk factors between ARDS and IPF [37], a situation that has turned pervasive in several complex diseases [48]. Another study reported variants of the advanced glycosylation end-product specific receptor (*AGER*) gene, encoding a marker of pulmonary epithelial lesions [38]. The authors also analysed the plasma levels of the soluble receptor for advanced glycation end-products (sRAGE) and endogenous secretory RAGE (esRAGE), observing that one *AGER* gene variant was associated with an increased risk of ARDS and higher plasma concentrations of sRAGE. Thus, the study suggested that an elevated plasma concentration of sRAGE in ICU patients could identify those who are more likely to be at risk for ARDS. Nevertheless, confirmatory studies to validate this association and to evaluate the functional role of the *AGER* variant are needed [38]. Likewise, it is believed that platelets influence the pathogenesis of ARDS through their role in the inflammatory responses and the dissemination of intravascular coagulation [49,50]. In fact, the decrease in platelet count after ICU admission could affect the prognosis of ARDS patients [51]. Of note, a genetic variant of the leucine-rich repeat–containing 16A (*LRRC16A*) gene, which has a role in platelet formation, was recently found to be associated with reduced ARDS risk [39]. On the other hand, since the mitogen-activated protein kinase 1 (*MAP3K1*) regulates numerous intracellular signalling pathways involved in inflammation and apoptosis, Morrell and colleagues hypothesized that there may be genetic variants of *MAP3K1* that can modify self-mediated changes in inflammation and transcriptional regulation and that can be associated with ARDS [40]. They identified a variant significantly associated with ventilator-free days and increased 28-day mortality. This variant was also associated with increased IL-1β, IL-6, IL-8, monocyte chemoattractant protein 1 and TNF-α production in ex-vivo stimulation of peripheral blood cells [40]. Additionally, Hernandez-Pacheco and colleagues performed an integrative multiomics analysis with data from rat models and ICU patients and identified a variant in the gene encoding the main VEGF receptor (*FLT1*) as a novel ARDS risk factor [41], suggesting that altered levels of this receptor could contribute to protection from lung injury, reducing the activity of VEGF and the vascular permeability in patients with ARDS [41,52]. One important contribution from this study is that it demonstrated that prioritization of genes with reproducible associations with ARDS was possible by integrating public data from transcriptomics in animal models and clinical samples, as well as from association studies [41]. Besides, it has been shown that elevated levels of soluble plasma forms of the FLT1 protein (sFLT1) may be related to the severity of sepsis, organ dysfunction and mortality of ICU patients [53,54]. In another study, the association between variants of the interleukin-17 (*IL17*) gene and the risk and prognosis of ARDS were analysed comparing ARDS patients with patients at risk [42]. In this study, the authors observed that two functional polymorphisms of *IL17* were associated with significant risk and prognosis of ARDS among East Asians [42]. Finally, a recent study reported the association of defensin beta 1 (*DEFB1*) gene variants on ARDS susceptibility and survival [43]. Carriers of the G allele at rs1800972 were more likely to develop ARDS and to have an unfavourable prognosis. That study concluded that a variant affecting gene transcription and posttranscriptional RNA stability of *DEFB1* was associated with ARDS risk and worse prognosis [43]. Overall, despite a paucity of candidate gene studies in ARDS and the inherent limitations of this design, results so far have provided new hypothesis testing avenues where association with the disease is increasingly reinforced.

Genome-wide association studies (GWAS) (Table 1) have been able to reveal hundreds of gene variants involved in the susceptibility and outcome of many complex diseases. However, despite the demonstrated utility of GWAS for the identification of new disease genes, the application of GWAS to ARDS or its main risk factors (sepsis, trauma, etc.) has been limited. In addition, these studies usually reveal variants with mild effects and, therefore, they explain a small proportion of the disease. Only two GWAS in ARDS patients have been published to date [55,56]. Christie and colleagues focused on trauma-associated ARDS cases of European ancestry and conducted the first GWAS of ARDS, involving two stages of association studies followed by a third stage of analysis of expression quantitative trait loci (QTL) [55]. This study was the first to support the feasibility of multi-centre GWAS of ARDS. They also revealed that variants of the gene encoding the protein tyrosine phosphatase receptor type F polypeptide-interacting protein alpha-1 (*PPFIA1*) were involved in trauma-associated ARDS. *PPFIA1* encodes liprin alpha, a protein involved in cell adhesion, integrin expression and cell-matrix interactions. Therefore, the identified gene suggested a compelling mechanism to explain ARDS pathogenesis [55]. More recently, Bime and colleagues [56] identified the selectin P ligand (*SELPLG*) gene as a new susceptibility locus for ARDS based on a study including all-cause ARDS in African-Americans. They observed that *SELPLG* expression in lung tissue increased significantly both in ventilator-induced and LPS-induced lung injury in murine models compared to controls [56]. Although the prohibitive genome-wide significance threshold was not reached in any of these two GWAS of ARDS, they both were able to reveal previously unrecognized ARDS susceptibility genes. Further studies will be needed to evaluate the robustness of these findings and to provide mechanistic implications.

ARDS survival has not been assessed through a GWAS approach to date. However, two GWAS have been completed for sepsis survival whose findings have implications for ARDS. Rautanen and colleagues [57] analysed 28-day mortality in European adult patients admitted to ICUs with sepsis, severe sepsis, or septic shock due to pneumonia or intra-abdominal infection. They completed a GWAS in three independent European cohorts—GenOSept/GAinS (Genetics of Sepsis and Septic Shock in Europe), Vasopressin in Septic Shock Trial (VASST) and the Human Activated Protein C Worldwide Evaluation in Severe Sepsis (PROWESS). In their study, they identified a common variant in the *FER* gene that was associated with a reduced risk of death from sepsis due to pneumonia. The protein encoded by this gene is a member of the FPS/FES family of non-transmembrane receptor tyrosine kinases. It regulates the cell-cell adhesion and mediates the signalling from the cell surface to the cytoskeleton through growth factor receptors [57]. Based on this study, other researchers conducted a survival analysis showing that patients with the *FER* variant had a greater risk of 90-day mortality compared to non-carriers [44]. They postulated that the *FER* variant could serve as a prognostic factor for survival in patients with severe ARDS due to pneumonia [44]. However, *FER* association with sepsis survival was not replicated in an independent study [58], questioning whether these findings have clinical relevance for prevention, therapy or risk stratification of sepsis. An independent GWAS study [59] found 14 loci with suggestive evidence of association with 28-day mortality from sepsis in the genes encoding the vacuolar protein sorting 13 homologs A (*VPS13A*) and the cysteine rich secretory protein LCCL domain containing 2 (*CRISPLD2*), as well as an intergenic variant at 13q21.33 in an independent data set. The protein product of *CRISPLD2* is related to the innate immunity and it is one of the best-validated biomarkers in sepsis research. This protein is decreased in septic shock and it is associated with changes in procalcitonin levels [60]. Among the strongest signals in this GWAS, there was a missense and potentially deleterious variant located in *VPS13A*, which encodes a molecule that is involved in the control of protein cycling through the trans-Golgi network and with an important regulatory role in autophagic degradation [61] Importantly, this GWAS could not validate the results provided by Rautanen and colleagues, although they did not focus exclusively on sepsis due to pneumonia, which may explain the discrepancy [59].

Whole-genome and whole-exome sequencing (WES) studies are becoming key approaches to elucidate the genetic variants involved in human diseases (Table 1) [62]. However, only two small WES studies have been carried out in ARDS so far [63,64]. Lee and colleagues performed a WES study in 88 individuals with sepsis-induced ARDS. The selection of patients was based on “ventilator-free days” (VFD), so that the subjects with high VFDs were compared to those with low VFDs. The authors identified infrequent variants present in 6488 genes, among which they found a strong association of variants of the myosin light chain kinase (*MYLK*) gene with ARDS [63]. *MYLK* encodes a key element of the cytoskeleton with multiple roles in the alveolar-capillary barrier of the airways, which was previously related to ARDS in independent candidate-gene studies [32]. On the other hand, a WES study including samples from 96 sepsis-induced ARDS patients that were compared with data available in public databases from population controls, supported the association of variants in genes encoding class I (HLA-B) and class II molecules of the major histocompatibility complex (*HLA-DRB1*, *HLA-DQA1*, *HLA-DQB*1 and *HLA-DRB5*) [64], which are critically involved in the immune response. This study also identified variants in three genes associated with ARDS susceptibility and severity: the arylsulfatase D gene (*ARSD*), the XK blood group, Kell family related complex subunits, member gene 3 (*XKR3*) and zinc finger protein 335 (*ZNF335*) [64]. All WES findings in ARDS still await validation in independent studies.

## 4. Causal Inferences with Mendelian Randomization

Due to disease acuity and heterogeneity, the genetic contribution to ARDS is not immediately apparent. There are many intermediate features that may have a direct causal relationship with the risk of the disease and that could be considered therapeutic targets [65]. Mendelian Randomization (MR) analysis of intermediate features could help to develop precision medicine options for ARDS [66,67]. MR consists in the analysis of the genetic variation underlying an intermediate feature and modelling it as an exposure indicator to detect potential causal effects in ARDS (Table 1) [68]. While they are not hypothesis free studies, their main advantage is that they are less influenced by confounding or reverse causation than conventional observational studies [68,69]. There is one particular application of MR to ARDS. Vascular permeability plasma angiopoietin-2 (ANG2) was suggested to be a strong ARDS biomarker [70,71,72]. An MR analysis was performed in The Molecular Epidemiology of SepsiS in the ICU (MESSI) cohort, which included data from 703 septic patients with measures of ANG2 in plasma at the time of admission to the ICU [45]. In that study, the authors confirmed that ANG2 levels were strongly associated with ARDS. They also showed that five variants of the *ANGPT2* gene were associated with ANG2 plasma levels among septic patients of European ancestry. Additionally, the strongest risk variant of *ANGPT2* in determining ANG2 levels was associated with an increased risk of ARDS, suggesting that ANG2 in plasma may be a causative factor in the development of ARDS [45]. More MR studies are needed to further understand the biology of ARDS and to identify discrete subgroups of ARDS, which would facilitate the development of precision therapies [73]. In this context, GWAS of outcome variables and endotypes and assessments in very large sample sizes remain to be tested in ARDS. Other genetic approaches, such as admixture mapping or whole-genome sequencing analyses, could help to identify causative factors of ARDS in future MR studies.

## 5. Transcriptomics

The evaluation of gene expression levels is another approximation to illustrate the physiological responses of multiple factors that contribute to the manifestation of ARDS (Table 1). Changes in the host transcriptome may also imply the role of the disordered host defence and inform about the disease progression [74]. Although most studies to date correspond to animal models, transcriptomic analysis based on microarrays and microRNAs have shown that sepsis and ARDS share many common biological processes. Acosta-Herrera et al. [52] analysed transcriptomics of lung tissues from sepsis animals with or without a severe lung injury, equivalent to ARDS and identified many common key mechanisms but at different levels of dysregulation. They also identified processes, such as lung development genes, that were uniquely altered among animals with lung injury. Peripheral blood transcriptomics in humans have shown a parallel scenario [75] since particular key mediators of the initial neutrophil response to infection, such as *LCN2*, *BPI*, *CD24*, *CASP1* and *MMP8*, were strongly dysregulated in patients with sepsis-associated ARDS compared to sepsis patients. These results show that there is a different but largely overlapping, gene response between ARDS and some of the main risk factors to develop it.

A recent study in lung tissues from animal models and in human lung cells used an integrative genomics approach to demonstrate a positive regulation of the C-type lectin domain family 4 member E (*CLEC4E*) gene and the CD300 molecule like family member F (*CD300LF*) during ARDS, which were previously detected by a transcriptome-wide association study (TWAS) (Table 1) [76]. Since *CLEC4E* had potential links to respiratory diseases, the authors suggested its possible role in the pathogenesis of ARDS [76]. An independent study detected a significant regulation of genes associated with essential protein functions, such as cyclin 1 (*CCNB1*) and cyclin 2 (*CCNB2*), which play a role in the cell cycle regulation and might be associated with the development of ARDS [77]. Likewise, another study compared the gene expression profile between patients with sepsis and with sepsis-induced ARDS [78] and identified differentially expressed genes related to cell cycle functions. In addition, after the construction of a protein-protein interaction network, the authors detected interesting targets such as CCNB1, CCNB2 and the DNA Topoisomerase II Alpha (TOP2A), which are important in cell cycle, transcription and replication of DNA. Although there is no direct evidence that cyclins are involved in the development of ARDS and given that mitosis can trigger cell apoptosis or lead to mutations, the study speculated that these molecules may play an unanticipated role in ARDS. In particular, it has been observed that DNA damage is related to acute lung injury and other pulmonary disorders [79]. In an array-based transcriptome analysis of peripheral blood cells stimulated with a Toll-like receptor 4 ligand ex vivo, it was observed an enrichment of inflammatory genes in subjects homozygous for a risk variant in *MAP3K1*, suggesting that this variant may predispose individuals to a more active inflammatory response [40]. In agreement with this finding, another study with microarray-based transcriptomics in peripheral blood from adult ARDS patients compared to septic patients showed that the most differentially expressed genes included key mediators of the initial neutrophil response to infection [75]. Although not yet applied to ARDS patients, sequencing of human transcriptomes (RNA-seq) could complement these studies and the conventional diagnosis and even help making clinical decisions [80,81]. In fact, using RNA-seq for metagenomics (metatranscriptomics) in bronchoalveolar lavage samples from patients with ARDS and severe pneumonia for analysing the relative abundance of bacterial, fungal and viral species, provided a rapid diagnosis of the infectious agent in approximately 50 h [81]. With this approach, the pathogen *Chlamydophila psittaci*—which is not commonly included in the standard diagnostic microbiology, although it is linked to zoonotic infections—was identified in one of the patients. This study demonstrated the utility of these approximations for a timely clinical intervention, which has obvious applications for ARDS [81].

Small non-coding RNAs (such as microRNAs) exert regulatory functions by simultaneously activating or inhibiting the expression of a series of genes. As a result, microRNAs could serve as therapeutic targets, as biomarkers and as elements for a better understanding of the ARDS pathogenesis. It is clear that a stratification based on genetic and molecular tests is still needed to identify those patients at higher risk of worsening or fatal outcomes. Although limited in number, current trends have focused the attention on the use of microRNA expression profiles in human peripheral blood [30,82]. Few studies have reported that particular microRNA species, such as miR-454 [83] and miR-125b [84], might be involved in ARDS. Another study suggested that miR-221 and miR-27b showed differences between pulmonary and extra-pulmonary ARDS and that miR-26a and miR-27a predicted survival among pulmonary ARDS patients [85]. In this regard, Han and colleagues observed high levels of miR-155 and miR-146a in the plasma of patients with severe sepsis and sepsis-associated ARDS and that their plasma levels predicted 30-day mortality in ARDS patients with relatively accuracy (areas under the curve [AUC] of 0.78 and 0.73, respectively) [86]. In another study, three microRNA species (miR-181a, miR-92a and miR-424) were significantly associated with ARDS in two independent cohorts [87]. Additionally, in a statistical model that included the Lung Injury Prediction Score, the authors obtained a subtle increase of the predictive capacity of the model (AUC from 0.70 to 0.72). While these values are still far from having clinical implications, the combination of biomarkers with clinical information and physiological measures is another promising path for improving the risk prediction in the context of ARDS [87]. Furthermore, mechanistic studies are necessary to link the role of these microRNA species with the modulation of molecular processes during ARDS [88].

## 6. Metagenomics

Since the lungs are not free of bacteria and the importance of a balanced lung microbiome has been shown to be central for the mucosal immunity, metagenomics is becoming an essential research and diagnostic tool for infectious diseases (Table 1). Lung microbiome composition is driven by different ecological principles that generate different interactions of the local microbiome and the immune system [89]. In the course of respiratory diseases, there is often a change in the lung bacterial community towards gamma-proteobacteria, containing many Gram-negative germs. While these bacteria benefit from the by-products of host inflammation, they also liberate inflammatory components, creating a potential cyclic mechanism. Attempts to leverage the microbiome alterations as a proxy for ARDS susceptibility or disease severity have just started. In a very elegant study assessing both a murine model of sepsis and clinical samples, Dickson and colleagues reported culture-independent evidence that the lung microbiome is enriched of gut bacteria during ARDS [90]. They sequenced part of the 16S ribosomal RNA (rRNA) and compared untreated and septic mice and observed that the sepsis group had greater number of detectable species in their lungs. Interestingly, bacterial communities in post-sepsis lungs were enriched with bacteria encountered in the murine gut (including members of the Bacteroidales order, *Enterococcus* species (sp.) and *Lachnospiraceae* sp.). This is consistent with transepithelial migration of new species into the lung during systemic inflammation [90]. They also performed bacterial metagenomics of 100 specimens of bronchoalveolar lavage fluid collected from patients with ARDS and compared those with healthy volunteers. They detected Bacteroides sp. and four anaerobic species abundant in the human gut (*B. fragilis*, *B. thetaiotaomicron*, *B. faecichinchillae* and *B. salyersiae*) that were undetectable via conventional culture techniques in the lung microbiome of ARDS patients [90].

In this context, integrated analyses of the host transcriptome and the microbial signatures (particularly if they are assessed with shotgun sequencing instead of targeted sequences of the 16S rRNA gene) will also have the potential to further improve our knowledge. While there are no published studies for ARDS yet, a recent study explored this avenue for IPF [74]. In IPF, these responses remained high in the longitudinal follow-up and differed between stable and progressive disease, suggesting that bacterial communities of the lower respiratory tract can act as persistent boost for the repetitive alveolar injury in IPF [74]. Those longitudinal changes could potentially be used as biomarkers at any stage of the disease process. Several other complementary longitudinal microbial studies are still needed to elucidate the interaction between the host and the microbes. Additionally, intervention studies with antibiotics or other measures capable of altering the microbiome could help to further determine the clinical relevance of these findings. As a final challenge, future microbiome research will be needed to determine if pulmonary microbiome alterations are related to the disease symptoms, how this can affect the lung homeostasis and if the pulmonary microbiome can be manipulated therapeutically [89].

## 7. Other Incipient Genomic Approaches

A recent study by Szilágyia and colleagues [91] evaluated the variation of methylation levels for the risk of ARDS (Table 1). In particular, they focused on the *MYLK* gene, which has an important regulatory role in endothelial barrier permeability in response to inflammation [92]. The authors compared methylation levels at several *MYLK* CpG sites between patients with ARDS and ICU controls. They observed several *MYLK* CpG sites associated with ARDS, with modifications by ethnicity of patients and a local cis-acting methylation QTL. These analyses can also contribute to increase our knowledge of molecular mechanisms and could explain the disparities observed among patients with ARDS [91]. Based on this, holistic epigenetic studies in ARDS patients have the promise to provide additional biomarkers and insights into ARDS pathogenesis.

Some studies have shown that levels of mtDNA in peripheral blood could be considered as a molecular pattern associated with damage [93] and that they could activate and initiate the early response of the immune system [94,95]. In this sense, Copies of mtDNA in peripheral blood were associated with 28-day mortality in ICU patients (Table 1) [96]. ARDS patients also had higher levels of circulating mtDNA. Despite these observations, the predictive capacity of peripheral mtDNA copies to serve for ARDS diagnosis or to predict ARDS prognosis has not been analysed yet.

## 8. Future Directions

**Figure 2:**
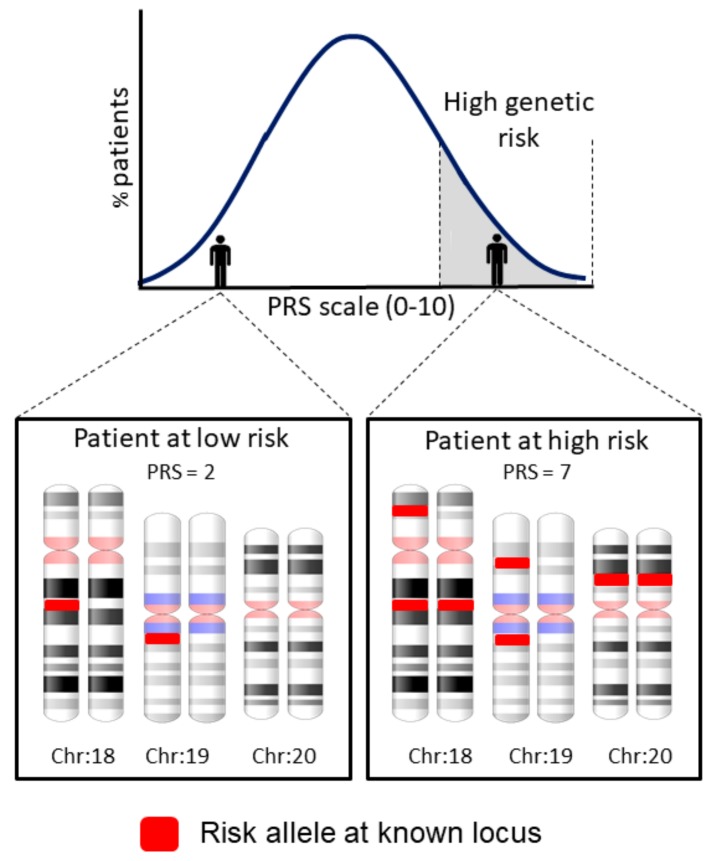
Schematic explanation of the polygenic risk scores (PRS), assuming the presence of five ARDS risk loci located in three chromosomes and a simplified interpretation of the genetic risk in the context of an intensive care unit (ICU) patient population.

Given the complexity of ARDS, the need to establish or improve predictive and prognostic scores is clear [5]. One approach that has the promise to become key for complex traits is the analysis of polygenic risk scores (PRS) (Table 1), which predict complex traits based on combined risk variants using a single score of risk burden (Figure 2).

In this way, PRS can define the individual genetic risk of a patient to develop the disease based on the combined genetic risk variants identified from published GWAS results [97]. PRS also allow to leverage the genetic overlap among diseases to reveal new disease genes [98]. However, although many genotype-trait associations are transferable among populations, the optimal choice of variants (and their weights) for extracting PRS models may differ from population to population [99]. For this reason, it is usually necessary to assess large sample sizes for testing the value of PRS [98].

GWAS of other outcome variables that are important in ARDS and of ARDS endotypes, as well as GWAS with larger sample sizes, will be needed to improve our understanding of the genetics of ARDS. Because most GWAS have been performed in Europeans, studies in diverse populations are necessary to identify more disease genes and to help to reduce health disparities [100,101]. Other related approaches based on the existing electronic medical records, such as the association of variants across diverse diseases and traits (the so called PheWAS), can help with the development of drugs or predict adverse drug events [102,103]. As such, PheWAS can be another powerful approach to reveal ARDS subtypes and to discover drugs to improve therapeutic possibilities.

Other approaches will provide complementary views to genetic associations studies in ARDS (Table 1) and elucidate new risks factors to be considered for PRS. Among them, admixture mapping analyses for allocating disease loci by leveraging the regional differences in the ancestry blocks across the genome of recently admixed populations and their correlations (co-inheritance) with disease loci [104] and whole genome sequencing (WGS) studies, will allow us to assess rare variants and other types of genetic variation beyond single nucleotide polymorphisms. Admixture mapping is a hypothesis-free approach that associates with a reduced proportion of false positive findings despite having a reduced penalty of statistical significance compared to GWAS. However, the main limitation is that this approach can only be applied to recently admixed populations where the evolutionary history is well known. With respect to WGS, it holds the promise for assessing genetic variation that remains obscure to other genomics approaches, which will be key for further identifying new disease genes, some of them likely involving unanticipated mechanisms in ARDS pathogenesis. Along with WGS, WES studies [105,106,107,108] will be also important to reveal rare variants with strong effects in the disease. These might be clinically relevant by providing sizeable improvements for risk stratification and links with other less prevalent pathologies but also because some will have pharmacogenetics implications to assist in the guidance of therapies. Despite their importance, there are no WGS studies and there are only a few WES studies in ARDS. An important limitation of these two approaches is the current lack of standards for the statistical assessment of disease associations and for detecting structural variation. To complicate things further, the computational requirements and the required infrastructure for conducting large WGS/WES studies are still out of reach for many laboratories.

## 9. Conclusions

There is no doubt that the use of new genomic approximations will allow to identify predictors for earlier and more precise characterization of ARDS and its risks. These predictors will help to improve the prognosis of patients and to define more effective treatments and diagnostic methods. Future studies should continue pursuing the aim to better stratify ICU patients by leveraging information from different omics.

## Figures and Tables

**Table 1:** Summary of the main genomic approaches applicable to acute respiratory distress syndrome (ARDS).

Approach	Aim	Main Advantages	Main Limitations	Phenotypes Assessed
Candidate-gene association study	To identify the statistical association between genetic variants for pre-specified genes of biological interest and the trait.	Simple approximation not requiring computational skills. Hypothesizes causality of the analysed variant Reduced penalty of statistical significance.	Non-reproducibility of the findings in independent studies complicating the interpretation.	Susceptibility, outcomes
Genome-wide association study (GWAS)	To identify the statistical association between genetic variants assessed across the genome and the trait.	Hypothesis-free approach. Allows to identify new pathogenic mechanisms, potentially leading to new therapeutic targets. Reduced proportion of false positives.	Many of the genes that are identified do not yet have a known biological implication in the trait. Large penalty of statistical significance. Large proportion of false negatives.	Susceptibility
Whole-exome sequencing (WES)	To identify the statistical association between genetic variants assessed across exons of all genes (exome) and the trait.	Same as indicated for GWAS. Allows analysis of rare and common genetic variants.	Blind to genetic variation occurring in the regulatory regions of genes. There is no standardization of the statistical tests. Requires advanced computational skills and dedicated infrastructure. More expensive than GWAS and candidate-gene studies for a fixed sample size.	Susceptibility, outcomes
Transcriptome-wide association study	To identify genomic loci associated with gene expression alterations related to the trait.	Same as indicated for GWAS.	Same as indicated for GWAS.	Susceptibility
Transcriptomics	To assess alterations of the gene expression and biological pathways in disease states focusing on particular targets or using array or sequencing-based approaches.	Allows to quantify and provides precise expression levels of genes simultaneously. A variant focusing on small non-coding species is possible. If sequencing-based, it allows the distinction of isoforms and allelic expression. If sequencing-based, it allows to map transcribed regions. If sequencing-based, it allows to evaluate gene expression levels in single cells.	The RNA isolation and handling require specialized materials and skills. If sequence-based, requires abundant RNA species (e.g., rRNA) to be depleted. Effects of this on the profiles are yet unknown. If sequencing-based, requires advanced computational skills and dedicated infrastructure. If sequencing-based, there is a lack of standardization of the optimal read depth.	Susceptibility and outcomes (array-based only)
Mendelian randomization	To assess the causality of a risk factor on a trait based on genetic predictors of the former.	Less affected by confusion or inverse causality.	Depends on many assumptions that need to be assessed for plausibility. Genetic predictors of the risk factor need to be known from previous studies.	Susceptibility
DNA methylation	To identify methylation levels at genomic loci associated with the trait.	Allows to quantitatively evaluate environmental exposures at DNA level. Permits the evaluation of functional effects of identified elements.	There is no standardization of the statistical tests. Necessity to control for collection tissues, environmental exposures and other relevant variables that affect the results.	Susceptibility
Metagenomics	To assess the collective microbial composition and function of environmental samples from genomic data.	Allows to characterize microbial communities (abundance, diversity and distribution) and deduce function without culturing. Allows to detect uncultivable microbes. With sufficient resolution, it allows to recover antibiotic resistance genes and virulence factors.	The same as indicated for DNA methylation. Requires advanced computational skills and dedicated infrastructure.	Susceptibility
Whole-genome sequencing	To identify the statistical association between genetic variants assessed across the genome and the trait.	Same as indicated for WES. Allows the better analysis of structural variation and variation in non-exonic regions of the genome.	There is no standardization of the statistical tests. Requires advanced computational skills and dedicated infrastructure. More expensive than WES studies for a fixed sample size.	None
Admixture mapping	To identify genomic regions that are associated with a trait based on ancestry markers.	Hypothesis-free approach. Reduced proportion of false positives. Reduced penalty of statistical significance.	Can only be applied in recently admixed populations and the evolutionary history must be known. Large proportion of false negatives. Identified loci at Mb resolution. There is no standardization of the statistical tests.	None
Polygenic risks	To stratify disease risks based on the cumulative effects of genetic variants.	Allows to stratify the risk with a single score. Allows to assess the genetic overlap among traits.	Genetic risk variants need to be known from previous studies. Difficulties in the transferability among populations.	None
Mitochondrial DNA levels	To assess its potential as a biomarker for a trait.	Simple approximation not requiring computational skills. May offer improvements for diagnostic or prognostic scores. Inexpensive approach.	Difficulties to reach optimal sensitivity and specificity. Strong dependency on sample collection and handling.	None

**Table 2:** Candidate genes associated with ARDS susceptibility or outcomes between December 2015 until April 2019.

Gene	Chr	Position (hg19)	rsID	Phenotype	Sample (Case/Control)		Population	Study
					Discovery	Validation		
*EGLN1*	1	231542656	rs516651	Outcome	264 *	--	European	Dötsch et al. [36]
*MUC5B*	11	1241221	rs35705950	Susceptibility	234/669	--	Multi-ethnic	Rogers et al. [37]
*AGER*	6	32151693	rs2070600	Susceptibility	59/405	--	Multi-ethnic	Jabaudon et al. [38]
*LRRC16A*	6	25426768	rs9358856	Outcome	414 *	--	Multi-ethnic	Wei et al. [39]
*MAP3K1*	5	56177743	rs832582	Outcome	306 *	241 *	European	Morrell et al. [40]
*FLT1*	13	28993669	rs9513106	Susceptibility	225/899	661/234	European	Hernandez-Pacheco et al. [41]
*IL17*	6	52185695	rs8193036	Susceptibility/Outcome	210/210	--	East Asian	Xie et al. [42]
		52186235	rs2275913					
*DEFB1*	8	6877901	rs1800972	Susceptibility	300/240	--	European	Feng et al. [43]
*FER*	5	108402140	rs4957796	Outcome	27/68	--	European	Hinz et al. [44]
*ANGPT2*	8	6370320	rs2442630	Susceptibility	178/226	--	European	Reilly et al. [45]
		6386620	rs2442608					

* Case-only study.
